# Fibrillin-Related Proteins Control Calcium Homeostasis in Dystrophic Muscle Across Species

**DOI:** 10.17912/micropub.biology.001697

**Published:** 2025-09-04

**Authors:** Damiano Marchiafava, Andres Vidal Gadea

**Affiliations:** 1 School of Biological Sciences, Illinois State University, Normal, Illinois, United States

## Abstract

Duchenne muscular dystrophy (DMD) involves progressive muscle degeneration associated with impaired calcium homeostasis, particularly defective calcium clearance during muscle relaxation. However, the mechanisms linking extracellular matrix (ECM) integrity to calcium regulation remain unclear. We investigated whether MUA-3 , a *Caenorhabditis elegans* fibrillin-related ECM protein, contributes to calcium dysregulation in dystrophic muscle. Using fluorescent calcium imaging in transgenic worms expressing muscle-specific GCaMP2, we found that *mua-3* downregulation selectively elevated resting calcium levels in healthy muscle, phenocopying the dystrophic calcium signature. Critically, partial *mua-3* downregulation had no additional effect in dystrophic ( *dys-1* ) muscle, where *mua-3* expression was already reduced by 57%, suggesting loss of *mua-3* function contributes to dystrophic pathology. In human dystrophic myoblasts, we observed parallel findings: elevated sarcoplasmic calcium concurrent with significant downregulation of fibrillin genes *FBN1/FBN2* . These findings identify fibrillin-related proteins as potential regulators of muscle calcium homeostasis across species and suggest that ECM-calcium coupling represents a conserved pathological mechanism in muscular dystrophy.

**Figure 1. Loss of dystrophin contributes to MUA-3/FBN1/FBN2 dysfunction in muscle:**
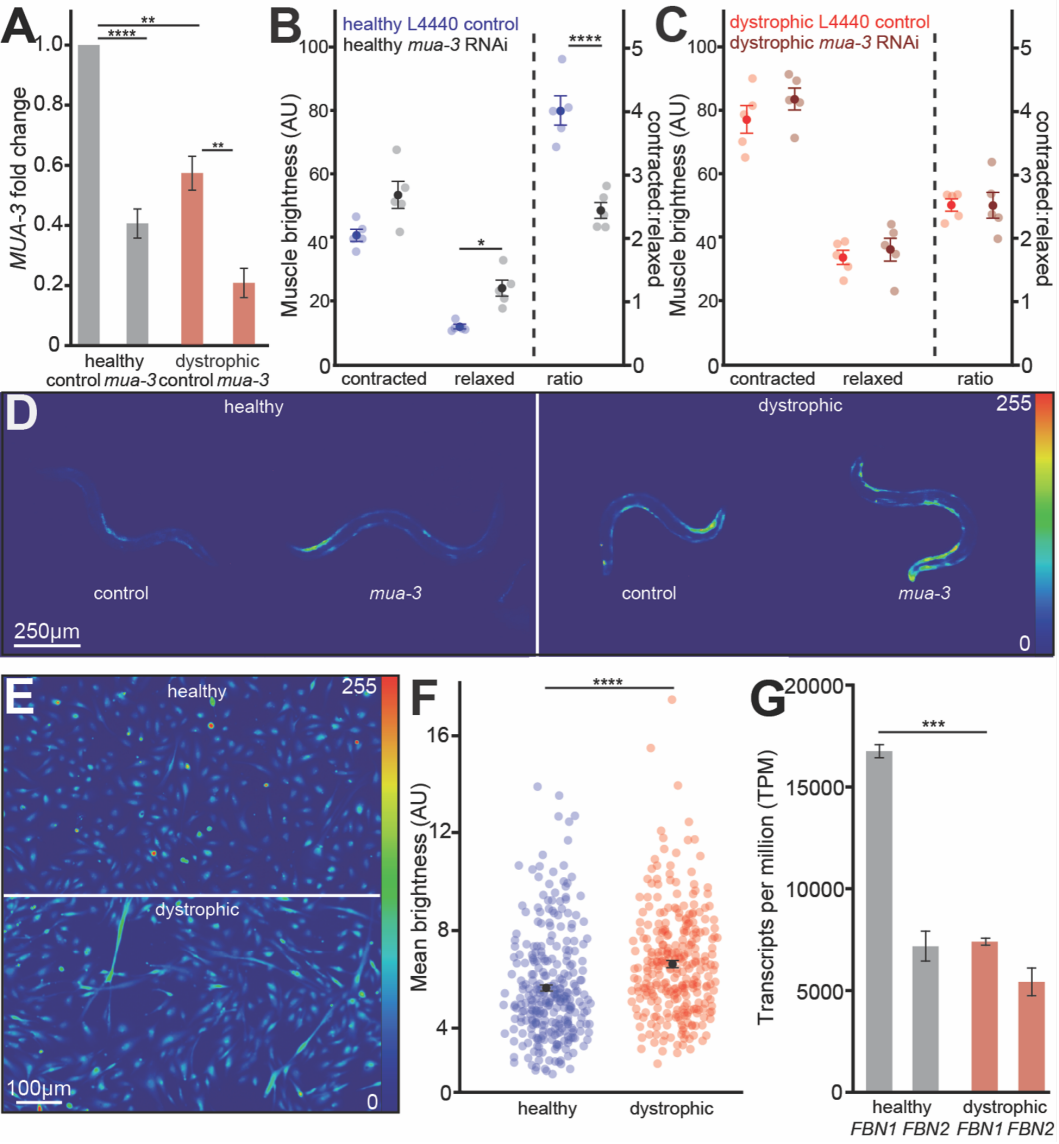
**A)** Dystrophic ( AVG6 ) worms display a 57% reduction in *mua-3* expression compared to healthy ( ZW495 ) worms. Healthy and dystrophic worm strains show reduced *mua-3* expression via RNAi by 59 and 62%, respectively, compared to their L4440 empty vector treated controls, measured via qRT-PCR. Fold change was calculated using the ΔΔCt method. (Tukey's Honest Significance Test, n=5 biological replicates). While **B)** downregulation of *mua-3* in healthy worms increases relaxed muscle brightness and decreases the contracted:relaxed brightness ratio, **C)** downregulation of *mua-3* in dystrophic worms does not. Brightness was calculated as (muscle fluorescence - background)/background for a given contracted (VL14 or VR14) or relaxed (DL13, DL15 or DR13, DR15) muscle (unpaired two-tailed t-test, n=5 biological replicates, 10 worms per replicate). **D)** Representative images of brightness levels of day-one adult wildtype ( ZW495 ) and dystrophic ( AVG6 ) worm strains expressing the calcium indicator GCaMP2 under the *Pmyo-3* promoter. Strains were raised feeding either an L4440 empty vector (control) or RNAi targeting *mua-3* . Brightness levels were linearly adjusted together for each image to enhance the visualization of each worm. **E)** Representative images depicting the brightness levels of healthy AB1190 and dystrophic AB1071 myoblasts when incubated in the Fluo 4-AM calcium indicator dye after 1 day of differentiation. Brightness levels were linearly adjusted together to enhance visualization. **F)** Dystrophic day-one differentiated myoblasts display elevated brightness levels compared to healthy myoblasts, measured via Fluo 4-AM. Brightness levels were assessed as the mean brightness level of each myoblast (unpaired two-tailed t-test, n=300). **G)** *FBN1* and *FBN2* are downregulated in undifferentiated dystrophic myoblasts compared to healthy myoblasts, measured via RNASeq (Wald test, n=6). *=p<0.05, **=p<0.01, ** *=p<0.001 ****=p<0.0001.

## Description

Duchenne Muscular Dystrophy (DMD) is a severe X-linked neuromuscular disorder affecting 1 in 5,000 male births, characterized by progressive muscle degeneration and weakness. DMD results from loss-of-function mutations in the dystrophin ( *DMD* ) gene, eliminating dystrophin, a critical cytoskeletal protein that connects the intracellular actin cytoskeleton to the extracellular matrix (ECM) via the dystrophin-associated protein complex (DAPC) (Duan et al., 2021). Loss of dystrophin destabilizes the DAPC and compromises sarcolemmal integrity, rendering muscle fibers susceptible to contraction-induced damage (Allen et al., 2016). This mechanical vulnerability initiates a cascade of pathological events including sarcolemmal tears, dysregulated calcium influx, proteolytic enzyme activation, and eventual muscle fiber necrosis (Mareedu et al., 2021).

Calcium dysregulation is central to dystrophic pathology. During normal muscle function, cytosolic calcium rises rapidly during contraction through ryanodine receptor activation, then must be efficiently cleared during relaxation primarily through SERCA pump activity. We previously demonstrated in *Caenorhabditis elegans* dystrophin ( *dys-1* ) mutants that this calcium clearance mechanism is specifically impaired, resulting in persistently elevated calcium during muscle relaxation (Hughes et al., 2019). By comparing calcium dynamics in both contracted and relaxed muscle states, we found evidence suggesting that the rise in intracellular calcium in dystrophic muscle is not due to excessive calcium entry but rather insufficient calcium removal. However, the molecular mechanisms linking loss of dystrophin to impaired calcium clearance remain incompletely understood.

The extracellular matrix provides critical mechanical support and signaling cues to muscle fibers. In *C. elegans* , the gene *mua-3* encodes a transmembrane protein essential for muscle-ECM attachment that shares structural and functional properties with the human fibrillin-1 ( FBN-1 ) and fibrillin-2 (FBN2) proteins (Davis & Summers, 2012; Ramirez & Sakai, 2010). The MUA-3 protein localizes to hemidesmosomes where body wall muscles attach to the hypodermis, and mutations in *mua-3* lead to progressive muscle detachment (Bercher et al., 2001; Fotopoulos et al., 2015). Given that both MUA-3 and human fibrillins are essential for maintaining ECM integrity and mechanical coupling, we hypothesized that MUA-3 dysfunction contributes to the calcium dysregulation observed in dystrophic muscle.

Here, we test whether *mua-3* regulates muscle calcium homeostasis and whether this function is compromised in dystrophic muscle. We predict that if *mua-3* contributes to calcium homeostasis, its downregulation should elevate resting calcium levels in healthy muscle. Furthermore, if *mua-3* function is already impaired in dystrophic muscle, additional downregulation should have a reduced further effect. Using parallel approaches in *C. elegans* and human dystrophic myoblasts, we demonstrate that fibrillin-related proteins are critical regulators of muscle calcium homeostasis and that their dysfunction contributes to dystrophic pathology across species.

To determine whether *mua-3* function might be compromised in dystrophic muscle, we first quantified *mua-3* expression levels in healthy ( ZW495 ) and dystrophic ( AVG6 ) day-one adult worms using qPCR. Dystrophic animals displayed a 57% reduction in *mua-3* expression compared to healthy controls ( Fig. 1A, p<0.01). To investigate the functional consequences of reduced *mua-3* , we used RNAi feeding to further downregulate *mua-3* expression. The RNAi clone ( K08E5.3 from the Ahringer library, sequence-verified in Supplementary File 1) effectively reduced *mua-3* expression by ~59% in healthy worms and ~62% in dystrophic worms relative to their respective L4440 empty vector controls ( Fig. 1A ). These results establish that dystrophic muscle has intrinsically reduced *mua-3* expression, which can be further decreased through RNAi.

To understand how reduced *mua-3* affects muscle calcium dynamics, we performed calcium imaging in worms expressing the fluorescent calcium indicator GCaMP2 specifically in body wall muscles under the *myo-3* promoter. We measured calcium levels during both muscle contraction (when calcium enters through ryanodine receptors) and relaxation (when calcium should be cleared by SERCA pumps), as well as calculating the contracted:relaxed ratio as a measure of calcium clearance efficiency (Hughes et al., 2019).

In healthy worms, *mua-3* downregulation did not affect peak calcium levels during muscle contraction ( Fig. 1B, left panel, p=0.31). However, it significantly increased calcium levels during muscle relaxation ( Fig. 1B, middle panel, p<0.001), resulting in a decreased contracted:relaxed ratio ( Fig. 1B, right panel, p<0.0001). Notably, the elevated relaxation calcium levels in *mua-3* RNAi-treated healthy worms (32.5 ± 3.2 AU) were comparable to those observed in dystrophic controls (35.1 ± 3.8 AU), suggesting that *mua-3* downregulation phenocopies the dystrophic calcium clearance defect.

In contrast, *mua-3* downregulation in dystrophic worms had no significant effect on calcium levels during contraction ( Fig. 1C, left panel, p=0.43), relaxation ( Fig. 1C, middle panel, p=0.51), or the contracted:relaxed ratio ( Fig. 1C, right panel, p=0.38). The lack of additional calcium accumulation upon *mua-3* downregulation in dystrophic muscle, despite successful RNAi-mediated knockdown, suggests that MUA-3 function is already compromised in the dystrophic background.

Representative images illustrating these calcium dynamics are shown in Figure 1D, with brightness adjusted uniformly across all images to facilitate visualization.

To determine whether the relationship between fibrillin dysfunction and calcium dysregulation is conserved across species, we examined human dystrophic myoblasts. Using Fluo-4 AM calcium indicator dye, we quantified sarcoplasmic calcium levels in age-matched healthy (AB1190) and dystrophic (AB1071) myoblasts after one day of differentiation. Dystrophic myoblasts displayed significantly elevated calcium levels (6.2 ± 0.3 AU) compared to healthy controls (4.1 ± 0.2 AU) ( Fig. 1E- F, p<0.0001).

To examine whether human fibrillin expression parallels the *mua-3* downregulation observed in worms, we analyzed RNAseq data comparing healthy and dystrophic myoblasts (GEO accession GSE299627). Both *FBN1* and *FBN2* showed significant downregulation in dystrophic myoblasts ( Fig. 1G, *FBN1* : log2 fold change = -1.2, p<0.0001; *FBN2* : log2 fold change = -0.9, p<0.0001). This parallel downregulation of fibrillin-related proteins in both species with concurrent calcium elevation supports a conserved mechanism linking ECM integrity to calcium homeostasis.

Analysis of transcription factors known to regulate *mua-3* expression revealed coordinated changes in dystrophic muscle. The zinc-finger transcription factor *ztf-7* showed significant upregulation in dystrophic worms (Hrach et al., 2020). Remarkably, the human ortholog ZNF277 showed a similar trend toward upregulation in dystrophic myoblasts (p=0.098), suggesting a conserved transcriptional response to muscle stress. This coordinated regulation across species indicates that the fibrillin-calcium axis may be modulated through conserved transcriptional networks, offering potential therapeutic targets.

Our findings establish fibrillin-related proteins as critical regulators of muscle calcium homeostasis and demonstrate that their dysfunction contributes to dystrophic pathology across species. The observation that *mua-3* downregulation in healthy muscle phenocopies the calcium clearance defect of dystrophic muscle, while having no additional effect in dystrophic muscle where *mua-3* is already reduced, provides compelling evidence that MUA-3 dysfunction is integral to dystrophic calcium dysregulation rather than a secondary consequence.

The use of partial RNAi knockdown rather than complete genetic ablation proved particularly informative, as it better models the pathophysiological condition where fibrillin-related proteins are reduced but not absent. Previous studies using *mua-3* null mutations reported severe phenotypes including larval lethality and muscle detachment (Bercher et al., 2001), while hypomorphic alleles like *mua-3 ( rh195 )* with ~30% residual expression show milder, adult-onset phenotypes (Plenefisch et al., 2000). Our RNAi approach, achieving similar partial reduction, revealed specific calcium regulatory functions without the confounding severe structural defects of null mutations.

The specificity of the calcium clearance defect, affecting relaxation but not contraction phases, aligns with our previous identification of impaired SERCA function in dystrophic muscle (Hughes et al., 2019). We propose that fibrillin-related proteins maintain the structural integrity of the ECM-sarcolemma interface necessary for proper calcium pump localization or function. When this integrity is compromised through either dystrophin loss or fibrillin reduction, calcium clearance mechanisms fail, leading to persistently elevated resting calcium that drives further muscle damage.

The parallel findings in human dystrophic myoblasts strengthen the translational relevance of our work. The substantial downregulation of both *FBN1* and *FBN2* in dystrophic myoblasts, coupled with elevated sarcoplasmic calcium, mirrors the *C. elegans* phenotype and suggests this mechanism is evolutionarily conserved. Given that fibrillins organize ECM microfibrils and regulate TGF-β signaling, their loss likely contributes to both structural and signaling defects in dystrophic muscle.

Alternative mechanisms could contribute to these observations. Compensation through redundant ECM proteins might partially mask more severe phenotypes. Additionally, chronic calcium elevation itself might downregulate fibrillin expression through feedback mechanisms. However, the conservation of this relationship across species and the coordinated transcriptional changes argue for a direct functional relationship.

The identification of conserved transcriptional regulators ( *ztf-7* / *ZNF277* ) that are upregulated in dystrophic muscle of both species suggests an attempted compensatory response that ultimately proves insufficient. Future work should test whether enhancing this transcriptional program or directly supplementing fibrillin function could ameliorate calcium dysregulation in dystrophic muscle. Additionally, investigating the mechanistic link between fibrillin-mediated ECM integrity and SERCA function could reveal new therapeutic targets for muscular dystrophy.

In conclusion, we demonstrate that fibrillin-related proteins are essential for maintaining muscle calcium homeostasis, with their dysfunction contributing to the pathological calcium elevation characteristic of dystrophic muscle. This conserved mechanism linking ECM integrity to cellular calcium regulation represents a potential therapeutic target for muscular dystrophy and possibly other diseases involving ECM disruption.

## Methods

***C. elegans* strains and culture conditions**

Animals were maintained at 20°C on nematode growth media (NGM) agar plates seeded with *Escherichia coli* OP50 as previously described (Brenner, 1974). Strains used in this study are listed in Table 1. The AVG6 strain was generated by crossing ZW495 with BZ33 . All strains were obtained from the Caenorhabditis Genetics Center (CGC), supported by NIH Office of Research Infrastructure Programs (P40 OD010440).

**Table 1. *C. elegans* strains and human cell lines used in this study**

**Table:** 

**Strain/Line**	**Genotype**	**Source**
ZW495	*zwIs132 [myo-3p::GCaMP2 + lin-15 (+)] *	CGC
AVG6	*dys-1 ( eg33 ); zwIs132 [myo-3p::GCaMP2 + lin-15 (+)] *	This study
AB1190	*Healthy paravertebral immortalized myoblasts*	Myology Institute
AB1071	*DMD* ΔE45-52 paravertebral immortalized myoblasts	Myology Institute

**RNA interference**

RNAi was performed by feeding as previously described (Timmons, 2006). Individual adult worms were placed on NGM plates containing 1 mM IPTG and 50 μg/mL carbenicillin, seeded with HT115 *E. coli* expressing either L4440 empty vector (control) or dsRNA targeting *mua-3* (clone K08E5.3 from the Ahringer library). Clone identity was verified by sequencing (see extended data file). Adult worms were allowed to lay eggs on RNAi plates, and progeny were maintained on RNAi food throughout development. All experiments were performed on day-one adults that had been exposed to RNAi from embryo to day-one adulthood, when all assays were performed.

**Quantitative RT-PCR**

Total RNA was extracted from synchronized day-one adult worms (five biological replicates, 50 worms per replicate) using TRIzol reagent. cDNA was synthesized using SuperScript III reverse transcriptase (Thermo Fisher Scientific). qPCR was performed using PowerUp SYBR Green Master Mix (Thermo Fisher Scientific, Cat #A25742) on a QuantStudio 7 Real-Time PCR System (Applied Biosystems, v1.3).

Each 10 μL reaction contained 5 μL PowerUp SYBR Green Master Mix, 0.5 μM each primer, and 1 μL cDNA template (50 ng for target genes, 50 pg for reference gene). Primers for *mua-3* : forward 5'-AGTGTGGGGCAAATGAAGCT-3', reverse 5'-ATTCGTCCTGGTGCATTCGT-3'. The reference gene *pmp-3* was used for normalization. Thermal cycling: 50°C for 2 min, 95°C for 2 min, followed by 40 cycles of 95°C for 15 s, 60°C for 1 min, and 72°C for 30 s. Melt curve analysis was performed from 60°C to 95°C. Relative expression was calculated using the ΔΔCt method. Statistical analysis was performed using unpaired t-tests in SigmaPlot 14.

**Calcium imaging in *C. elegans***

Day-one adult animals expressing *myo-3p::GCaMP2* were placed on 2% agarose pads without anesthesia to preserve normal muscle activity. Animals were imaged using an Olympus SZX12 stereomicroscope equipped with a Flea2 camera (Point Grey) capturing at 30.3 Hz. Fluorescence excitation was provided by a Sola Light Engine with minimal exposure to prevent photobleaching. Image sequences (1032×776 pixels) were captured as worms performed natural crawling movements. All calcium imaging was performed exclusively on day-one adult animals to maintain consistency with qPCR experiments and avoid developmental variability.

**Calcium quantification**

ImageJ (v1.53) was used to measure GCaMP2 fluorescence intensity. For each worm, regions of interest (ROIs) were drawn around specific muscle cells. Contracted muscle was assessed at the maximum bend of the midbody during typical crawling motion, measuring ventral muscles (VL14 or VR14) on the concave side. Relaxed muscle was assessed on the opposite dorsal side (DL13, DL15 or DR13, DR15), where muscles are extended and calcium should be minimally elevated. This approach follows established methodology (Hughes et al., 2019) for detecting calcium clearance defects. Background fluorescence was measured from an adjacent area outside the worm. Brightness ratio was calculated as (muscle fluorescence - background) / background. The contracted:relaxed ratio was calculated by dividing contracted muscle brightness by relaxed muscle brightness for each animal. Fifty animals were analyzed per condition across five independent trials.

**Human cell culture**

Immortalized human skeletal myoblast lines from age-matched (16-year-old male) healthy (AB1190) and dystrophic (AB1071) donors were obtained from the Myology Institute, France. Cells were maintained in Skeletal Muscle Growth Media (PromoCell, Cat #C-23060) supplemented with 10% FBS on plates coated with 1% Matrigel (Corning, Cat #CB-40234A). All experiments used cells between passages 3-9 maintained at 37°C with 5% CO2.

**Calcium imaging in human myoblasts**

Myoblasts were seeded at 1×10^5 cells/mL in Matrigel-coated 12-well plates and differentiated for 24 hours in differentiation medium (2% horse serum in DMEM). Cells were loaded with 5 μM Fluo-4 AM (Thermo Fisher Scientific, Cat #F14201) in growth medium for 30 minutes at 37°C, washed three times with PBS, and imaged in FluoroBrite DMEM (Gibco, Cat #A1896701) using a Keyence BZ-X810 fluorescence microscope. Images were acquired from 10 random fields per well, with analysis of mean fluorescence intensity per cell using ImageJ. Three hundred cells were analyzed per condition across three independent experiments.

**RNA sequencing and analysis**

RNA was extracted from proliferating myoblasts at 40% confluence using DirectZol RNA Miniprep Kit (Zymo Research, Cat #R2050). RNA quality was verified by Bioanalyzer (RIN > 8.0). Library preparation and sequencing were performed by Azenta Life Sciences. mRNA was enriched using poly-A selection, and libraries were prepared using NEBNext Ultra II RNA Library Prep Kit for Illumina (NEB, Cat #E7770). Paired-end 150 bp sequencing was performed on an Illumina NovaSeq 6000, achieving >30 million reads per sample. Raw reads were aligned to the human genome (GRCh38) using STAR (v2.7.9). Differential expression analysis was performed using DESeq2 (v1.32.0) with Wald test for significance. Data are available at GEO accession GSE299627.

**Statistical analysis**

All statistical analyses were performed using SigmaPlot 14. For comparisons between two groups, unpaired two-tailed t-tests were used when data met normality assumptions (Shapiro-Wilk test); otherwise, Mann-Whitney U tests were applied. Sample sizes were determined based on power analysis from preliminary experiments. Significance levels: *p<0.05, **p<0.01, ***p<0.001, ****p<0.0001. Data are presented as mean ± SEM unless otherwise noted.

**Data availability**

All RNAseq data are publicly available in GEO (accession GSE299627, reviewer token: ubypeioctfgxrit). Original imaging data and analysis scripts are available upon request.

## Data Availability

Description: PlasmidSaurus sequence of mua-3 RNAi lasmid used in this study. Resource Type: Dataset. DOI: https://doi.org/10.22002/n34tb-9x767
